# Supplementary material for: Cellular and Genomic Instability Induced by the Herbicide Glufosinate-Ammonium: An In Vitro and In Vivo Approach
Source: Cells. 2024 May 24;13(11):909. doi: 10.3390/cells13110909 (PMC11172084; doi:10.3390/cells13110909)
Supplement: Supplementary file 1 [file cells-13-00909-s001.zip › cells-2991627-supplementary.pdf]

Supplementary Material Table S1 - Induction of micronuclei produced by Glufosinate ammonium in cultured human lymphocytes

Test substance (µg/mL)	N	MNi	MNi/N ± S.D. (‰)	NBUDs	NBUDs/N± S.D. (‰)	CBPI ± S.D.
NC	20,000	14	0.700±0.571	8	0.400±0.503	2.009±0.061
MMC (0.100)	20,000	67	3.350±1.226***	45	2.250±1.482***	1.445±0.057***
GLA (0.500)	20,000	58	2.900±1.021***	27	1.350±1.089	1.820±0.069***
GLA (0.200)	20,000	41	2.050±0.686***	20	1.000±0.858	1.874±0.033***
GLA (0.100)	20,000	36	1.800±0.696*	25	1.250±0.910	1.902±0.024***
GLA (0.050)	20,000	33	1.650±0.671	12	0.600±0.598	1.925±0.029***
GLA (0.020)	20,000	21	1.050±0.605	14	0.700±0.657	1.956±0.047**
GLA (0.010)	20,000	17	0.850±0.671	11	0.550±0.650	1.989±0,071

CBPI = Cytokinesis-Block Proliferation Index; GLA = Glufosinate Ammonium; MNi = micronuclei;
N = total number of scored binucleated cells; NBUD = nuclear buds; NC = Negative Control;
MMC = Mitomycin-C; S.D. = Standard Deviation.
*** $P<0.001$, with respect to negative control
* $P = 0.011$, with respect to negative control
** $P = 0.002$, with respect to negative control

Supplementary Material Table S2 - Induction of micronuclei produced by Glufosinate ammonium in hemocytes of *Lymnaea stagnalis*

Test substance (µg/mL)	N	MNi	MNi/N ± S.D. (‰)	NBUDs	NBUDs/N± S.D. (‰)	BNC	BNC/N± S.D. (%)
NC	20,000	20	1.000±0.649	21	1.050±0.686	19	0.950±0.686
GLA (0.500)	20,000	43	2.150±0.671***	48	2.400±0.681	34	1.700±0.733***
GLA (0.200)	20,000	36	1.800±0.769*	38	1.900±0.718	32	1.600±0.754*
GLA (0.100)	20,000	30	1.500±0.688	30	1.500±0.761	29	1.450±0.605
GLA (0.050)	20,000	23	1.150±0.587	28	1.400±0.598	29	1.450±0.686
GLA (0.020)	20,000	21	1.050±0.605	24	1.200±0.523	24	1.200±0.616
GLA (0.010)	20,000	21	1.050±0.605	23	1.150±0.587	22	1.100±0,641

BNC = binucleated cells; GLA = Glufosinate Ammonium; MNi = micronuclei; N = total number of scored hemocytes;
NBUD = nuclear buds; NC = Negative Control;
MMC = Mitomycin-C; S.D. = Standard Deviation.

MNi: *** $P<0.001$, with respect to negative control; * $P = 0.023$, with respect to negative control.
NBUDs: *** $P<0.001$, with respect to negative control; * $P = 0.016$, with respect to negative control.

Supplementary Materials Table S3 – Variation (mm) in shell growth at different GLA concentrations

N	NC	GLA 0.500 µg/mL	GLA 0.200 µg/mL	GLA 0.100 µg/mL	GLA 0.050 µg/mL	GLA 0.020 µg/mL	GLA 0.010 µg/mL
1	16.80	9.02	11.18	10.74	12.30	14.72	13.16
2	17.30	8.90	11.63	9.69	12.57	14.49	12.59
3	17.30	9.77	9.49	12.32	15.31	12.77	14.55
4	14.40	12.67	11.52	13.93	14.86	14.68	14.81
5	11.80	8.74	12.92	11.30	13.28	10.43	14.29
6	16.60	8.91	11.14	11.90	12.05	16.13	13.42
7	18.80	9.56	11.02	11.33	12.61	15.99	12.67
8	19.10	11.55	9.47	13.90	13.91	15.57	12.55
9	15.50	10.72	10.88	14.18	10.84	12.60	14.19
10	11.90	10.14	10.98	13.65	13.23	13.77	13.02
11	16.10	8.28	12.39	9.32	14.44	13.39	14.28
12	11.90	10.46	10.56	13.55	12.55	11.77	15.77
13	15.70	10.65	13.25	8.19	14.70	14.29	15.26
14	20.10	11.60	9.18	11.87	11.20	14.37	15.87
15	20.60	8.81	12.24	13.65	12.68	13.41	15.46
16	20.40	11.16	11.24	11.30	13.29	13.53	15.28
17	18.10	8.12	13.72	13.93	13.37	12.23	14.67
18	17.50	11.46	9.64	11.35	14.22	12.27	14.95
19	19.60	8.08	13.56	11.07	14.33	12.30	17.09
20	18.40	11.35	9.56	11.00	11.69	12.43	14.21
**Mean**	**16.90*** **	**10.00**	**11.28**	**11.91**	**13.17**	**13.55**	**14.40**
**S.D.**	**2.74**	**1.36**	**1.40**	**1.72**	**1.24**	**1.49**	**1.23**

GLA = Glufosinate ammonium; S.D. = Standard Deviation
*** P<0.001; significantly higher with respect to all GLA concentrations
